# Hidden in Plain Sight: Nocardia farcinica Posterior Shoulder Abscess Temporally Associated With Sports Massage in an Immunocompetent Host

**DOI:** 10.7759/cureus.113691

**Published:** 2026-07-30

**Authors:** Babar A Rashid, Ketan Kantamaneni, Mohamed Hashem

**Affiliations:** 1 Department of Trauma and Orthopedics, East Lancashire Hospitals NHS Trust, Blackburn, GBR; 2 Department of Orthopedics, East Lancashire Hospitals NHS Trust, Blackburn, GBR

**Keywords:** immunocompetent host, musculoskeletal nocardiosis, nocardia farcinica, shoulder abscess, soft tissue infection, sports massage, surgical drainage

## Abstract

*Nocardia farcinica* is an uncommon but clinically significant pathogen characterized by enhanced intrinsic antimicrobial resistance and a recognized propensity for hematogenous dissemination. Musculoskeletal nocardiosis is rare and most commonly affects immunocompromised individuals following hematogenous spread from a pulmonary source. We report a male patient with a prior history of curative esophagectomy for esophageal carcinoma who presented with a four-week history of progressive right posterior shoulder pain, systemic features of infection, and markedly elevated inflammatory markers following a vigorous sports massage, without documented immunosuppression, skin breach, or identifiable traumatic inoculation event. Magnetic resonance imaging demonstrated a complex fluid collection measuring approximately 53 × 55 × 45 mm centered within the right teres major region with extensive periscapular soft-tissue edema. Two sequential surgical drainage procedures were performed, collectively yielding substantial purulent material without evidence of glenohumeral joint involvement or osteomyelitis. Microbiological culture identified heavy growth of *N. farcinica*, necessitating cessation of empirical intravenous flucloxacillin and initiation of linezolid, followed by a planned six-month course of oral co-trimoxazole. Cross-sectional imaging excluded disseminated disease. The patient achieved satisfactory clinical improvement with preserved neurovascular function at follow-up. This case represents a potentially novel mechanism of nocardial inoculation via vigorous deep soft-tissue massage and underscores the importance of considering atypical pathogens in deep soft-tissue infections that follow an unexpected or treatment-refractory clinical course.

## Introduction

Nocardia species are aerobic, filamentous, weakly acid-fast Gram-positive actinomycetes found ubiquitously in soil, decaying organic matter, and water. Human infection most commonly arises through inhalation, resulting in primary pulmonary disease, or through direct cutaneous inoculation following disruption of the skin barrier, producing primary cutaneous or lymphocutaneous nocardiosis [1,2]. *Nocardia farcinica* is a clinically important species within the *Nocardia asteroides* complex, distinguished by greater intrinsic antimicrobial resistance compared with other nocardial species and a well-recognized propensity for hematogenous dissemination, particularly to the central nervous system [1,3].

Musculoskeletal nocardiosis, encompassing pyomyositis, septic arthritis, osteomyelitis, bursitis, and deep soft-tissue abscess formation, is an uncommon manifestation of nocardial disease predominantly reported in immunocompromised individuals [4,5]. Primary cutaneous and soft-tissue nocardiosis in immunocompetent individuals has been documented in the context of penetrating trauma, thorn injuries, insect bites, fractures, and environmental contamination, though deep-space infections involving the posterior shoulder girdle musculature are exceptionally uncommon [2,6-8].

We present a case of *N. farcinica* deep posterior shoulder abscess in a patient with no active immunosuppression and no documented skin breach, temporally associated with a vigorous sports massage. This temporal timeline was established through detailed history-taking, which revealed the immediate onset of localized swelling and tightness in the triceps and posterior shoulder directly following a 40-minute manual sports massage performed two weeks prior to admission. This case represents a potentially novel inoculation mechanism and highlights the diagnostic challenges inherent in atypical soft-tissue infections.

## Case presentation

A 62-year-old male patient presented to the emergency department with a four-week history of progressive right posterior shoulder pain. Approximately four weeks before admission, he experienced the onset of pain localized to the right posterior shoulder following a vigorous sports massage. Symptoms progressively worsened over the preceding days, accompanied by fever, rigors, and marked restriction of right shoulder movement. There was no history of acute trauma, skin breach, open wound, or penetrating injury to the region. However, further history regarding antecedent events revealed that the patient had undergone a 40-minute manual (hands-on) deep-tissue sports massage administered by a professional practitioner two weeks prior to symptom onset. The session focused primarily on the right triceps and posterior shoulder aspect, involving deep manual friction and repeated passive glenohumeral abduction stretches to elongate the joint structures. No invasive instruments, needles, cupping tools, or instrument-assisted devices were utilized. The patient denied paraesthesia, sensory loss, or weakness of the right upper limb.

Relevant past medical history included esophageal carcinoma treated by curative esophagectomy with gastric pull-up in 2023, essential hypertension, familial hypercholesterolemia, diverticular disease, sliding hiatus hernia, esophagitis, and myofascial pain syndrome. Regular medications comprised ramipril, amlodipine, lansoprazole, amitriptyline, codeine, ferrous fumarate, and a recent course of fluconazole. No drug allergies were documented.

On initial assessment at the Emergency Medicine department, the patient was alert and orientated with hemodynamic stability. Observations included a temperature of 36.5°C, a heart rate of 79 beats/minute, a respiratory rate of 18 breaths/minute, a blood pressure of 129/79 mmHg, and an oxygen saturation of 98% on room air. Inspection revealed significant swelling over the posterior aspect of the right scapula extending superiorly into the triceps region. The overlying skin was erythematous and warm, with intense tenderness on palpation, but without fluctuance or skin breach. Neurological examination of the right upper limb was intact without sensory deficit. Active range of movement demonstrated marked restriction: forward flexion to 60° (contralateral 150°), extension to 0° (contralateral 20°), abduction to 90° (contralateral 120°), and absent adduction (contralateral 20°). All movements exacerbated pain. Elbow, wrist, and hand movements were unaffected.

Laboratory investigations demonstrated leukocytosis with a white blood cell count of 20.8 × 10⁹/L (normal range: 4.0-11.0 × 10⁹/L) and a markedly elevated C-reactive protein (CRP) of 202 mg/L (normal range: <5 mg/L). Intravenous flucloxacillin 2 g four times daily and intravenous paracetamol were commenced. The general surgical team urgently reviewed the patient and identified features consistent with severe cellulitis with raised inflammatory markers and spiking pyrexia, raising concern for osteomyelitis or septic arthritis. Following multidisciplinary discussion, care was transferred to the trauma and orthopedics team.

Orthopedic assessment confirmed a four-week history of progressive right posterior shoulder pain, erythema, and swelling without glenohumeral joint effusion, acute trauma, or skin breach. Examination confirmed erythematous, warm, and swollen posterior shoulder and scapular tissues with intense tenderness but without fluctuance. Neurovascular examination remained intact with normal capillary refill and palpable distal pulses. The patient was admitted under orthopedics for intravenous antibiotics and close monitoring, with nil by mouth from the early hours of the very next day in preparation for potential surgical drainage.

**Figure 1:**
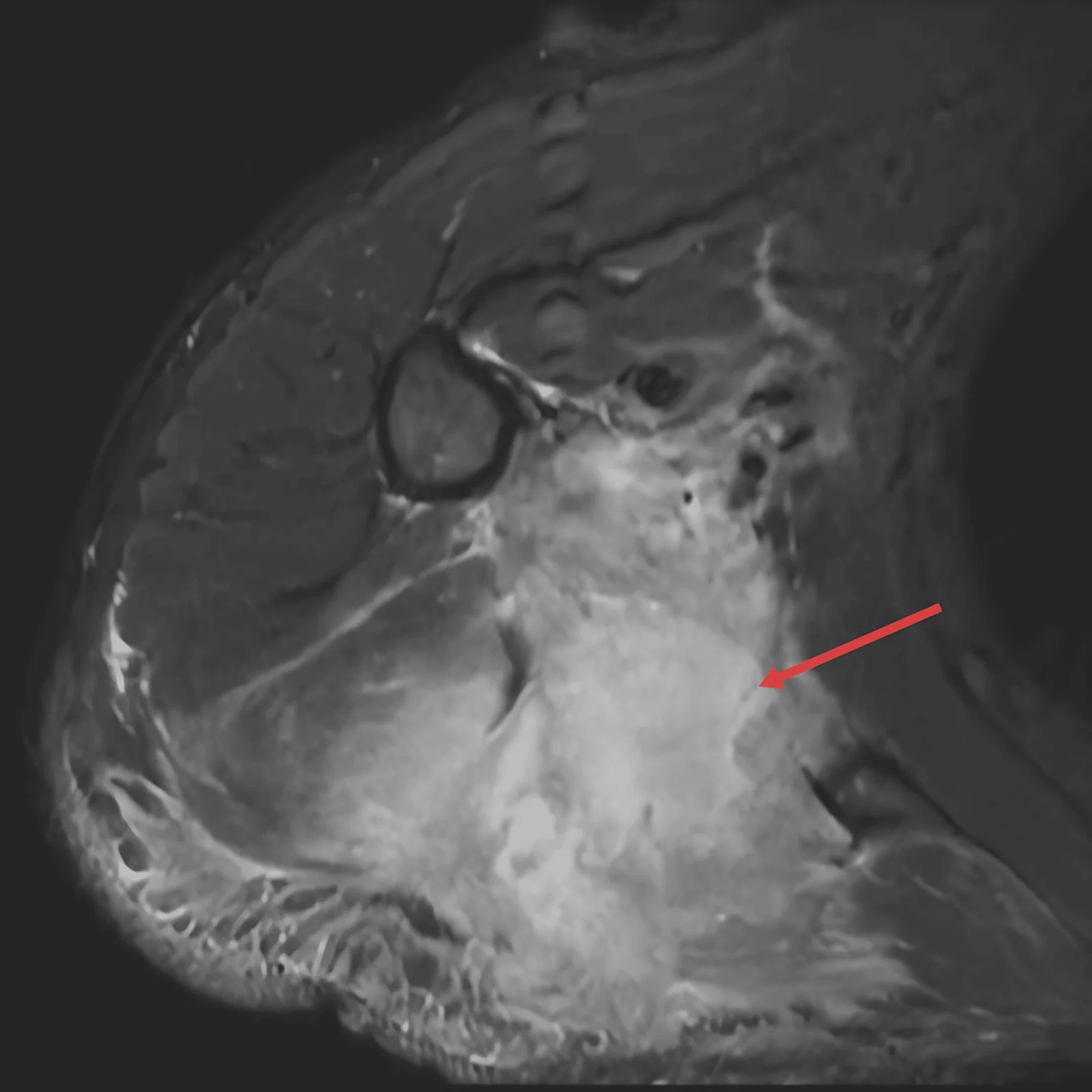
Preoperative MRI of the right shoulder demonstrating a complex fluid collection (arrow) centered on the right teres major region measuring 53 × 55 × 45 mm with extensive periscapular soft-tissue edema in the T2 axial view MRI: magnetic resonance imaging

**Figure 2:**
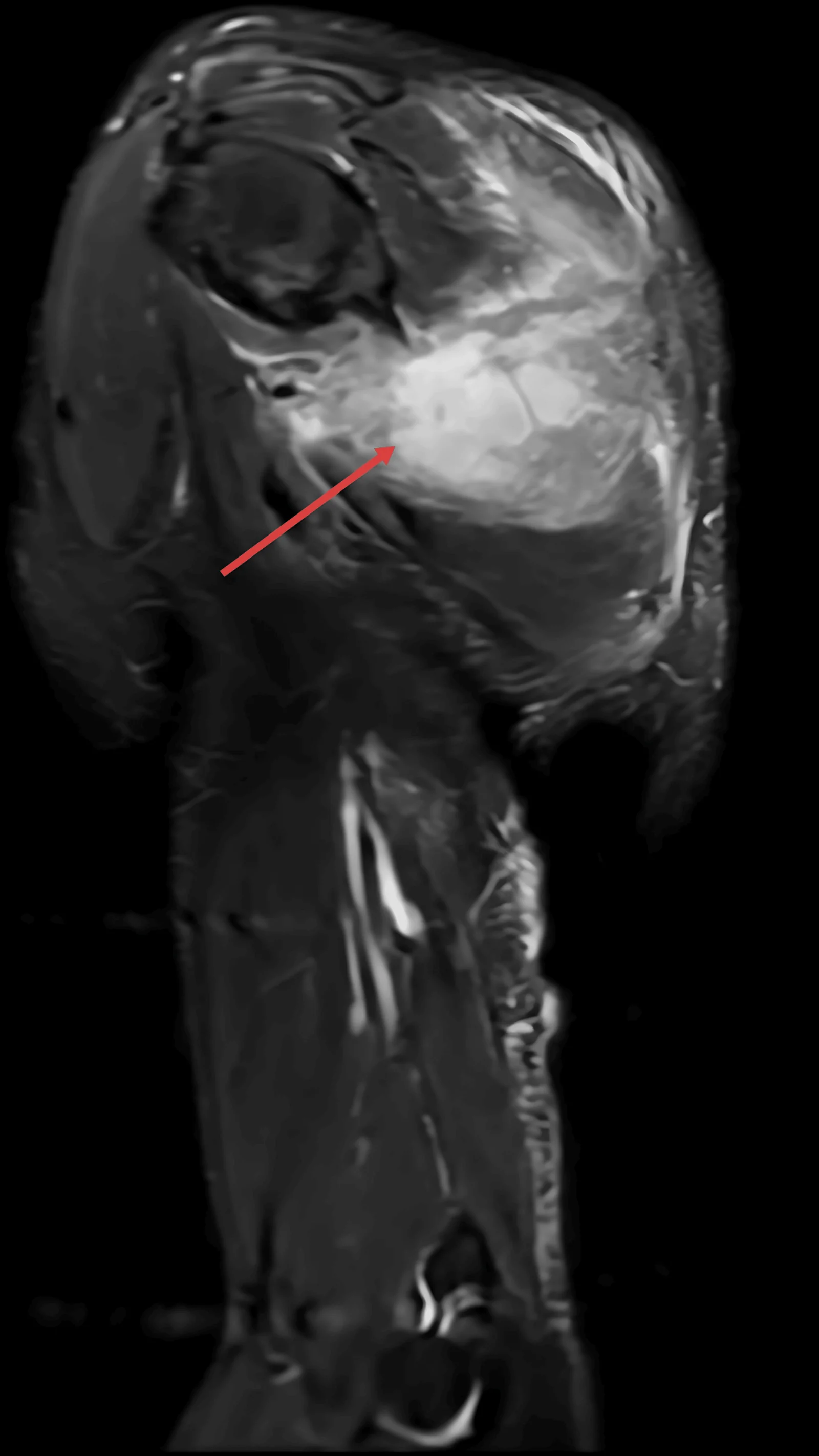
Preoperative MRI of the right shoulder demonstrating a complex fluid collection (arrow) centered on the right teres major region measuring 53 × 55 × 45 mm with extensive periscapular soft-tissue edema in the T2 sagittal view MRI: magnetic resonance imaging

A private magnetic resonance imaging (MRI) scan of the right shoulder performed a couple of days before admission, obtained for review, demonstrated mild-to-moderate acromioclavicular joint arthropathy with subacromial impingement features and tendinopathic rotator cuff changes including a partial-thickness articular surface tear of the infraspinatus tendon. Critically, MRI of the right upper arm identified a substantial complex fluid collection measuring approximately 45 mm craniocaudally and 53 × 55 mm in the transverse and anteroposterior planes, centered on the right teres major region. The collection demonstrated internal complexity, with extensive surrounding soft-tissue edema involving the deltoid, triceps, teres minor and major, infraspinatus, subscapularis, and latissimus dorsi muscles, with additional overlying lateral chest wall and subcutaneous edema. No sinister bone marrow changes or evidence of osteomyelitis was identified (Figures 1, 2).

Despite ongoing intravenous flucloxacillin, inflammatory markers remained elevated and pain persisted. Two days later, under general anesthesia in the lateral position, a 7-cm incision was made parallel and inferior to the inferior border of the infraspinatus muscle over the fluctuant region. Approximately 200 mL of frank purulent material was evacuated from a large abscess cavity without communication to the glenohumeral joint. Loculations were gently disrupted, curettage was performed, and a thorough saline washout was completed using a total of 2.5 L, followed by betadine irrigation. Purulent specimens were sent for microbiological culture and sensitivity. A subcutaneous drain was placed and secured, and wound closure was performed with vertical mattress sutures. Intravenous gentamicin was administered prophylactically on induction alongside ongoing flucloxacillin. Estimated blood loss was less than 200 mL, and the procedure was uneventful.

At ward review on the third day after the surgery, the drain had been removed without complication, and the wound was clean with no discharge on expression. However, CRP had risen to 223 mg/L, and temperature remained elevated at 38.3°C. Microbiological culture of operative specimens identified heavy growth of *N. farcinica*. Given this pathogen's resistance profile and recognized risk of dissemination, infectious diseases specialists were urgently consulted. Intravenous flucloxacillin was discontinued, and linezolid 600 mg twice daily was initiated, with further susceptibility testing requested.

On postoperative day 5, the patient demonstrated progressive clinical improvement with reduced swelling and improved pain control. Targeted screening for underlying infectious immunodeficiency and occult chronic infection returned negative results. Specific testing included a fourth-generation HIV antibody/p24 antigen immunoassay, hepatitis B surface antigen, hepatitis C antibody, cytomegalovirus (CMV) IgM antibody, and a QuantiFERON-TB Gold interferon-gamma release assay (tuberculosis (TB) nil: 0.02 IU/mL, TB1 Ag: 0.01 IU/mL, TB2 Ag: 0.01 IU/mL, mitogen control: 0.94 IU/mL), confirming no underlying retroviral infection, chronic viral hepatitis, acute CMV, or latent *Mycobacterium tuberculosis* infection. Microbiological review recommended follow-up within two weeks to determine treatment duration, noting that nocardiosis typically requires 6-12 months of antimicrobial therapy given the risk of dissemination. Computed tomography of the thorax, abdomen, and pelvis (CT TAP) was requested to exclude disseminated infection.

On postoperative day 6, a second incision and drainage procedure (relook) was performed due to persistent cavity dimensions and concern for residual infection. Approximately 80 mL of purulent material was drained, with blunt dissection to clear both anterior and superficial posterior cavity components. Necrotic tissue was excised, 3 L of saline washout was performed, and the wound packed with Betadine-soaked gauze. All sutures were removed at this procedure. Microbiological specimens obtained at the second procedure yielded no growth. The patient was maintained on intravenous antibiotics with close monitoring.

**Figure 3:**
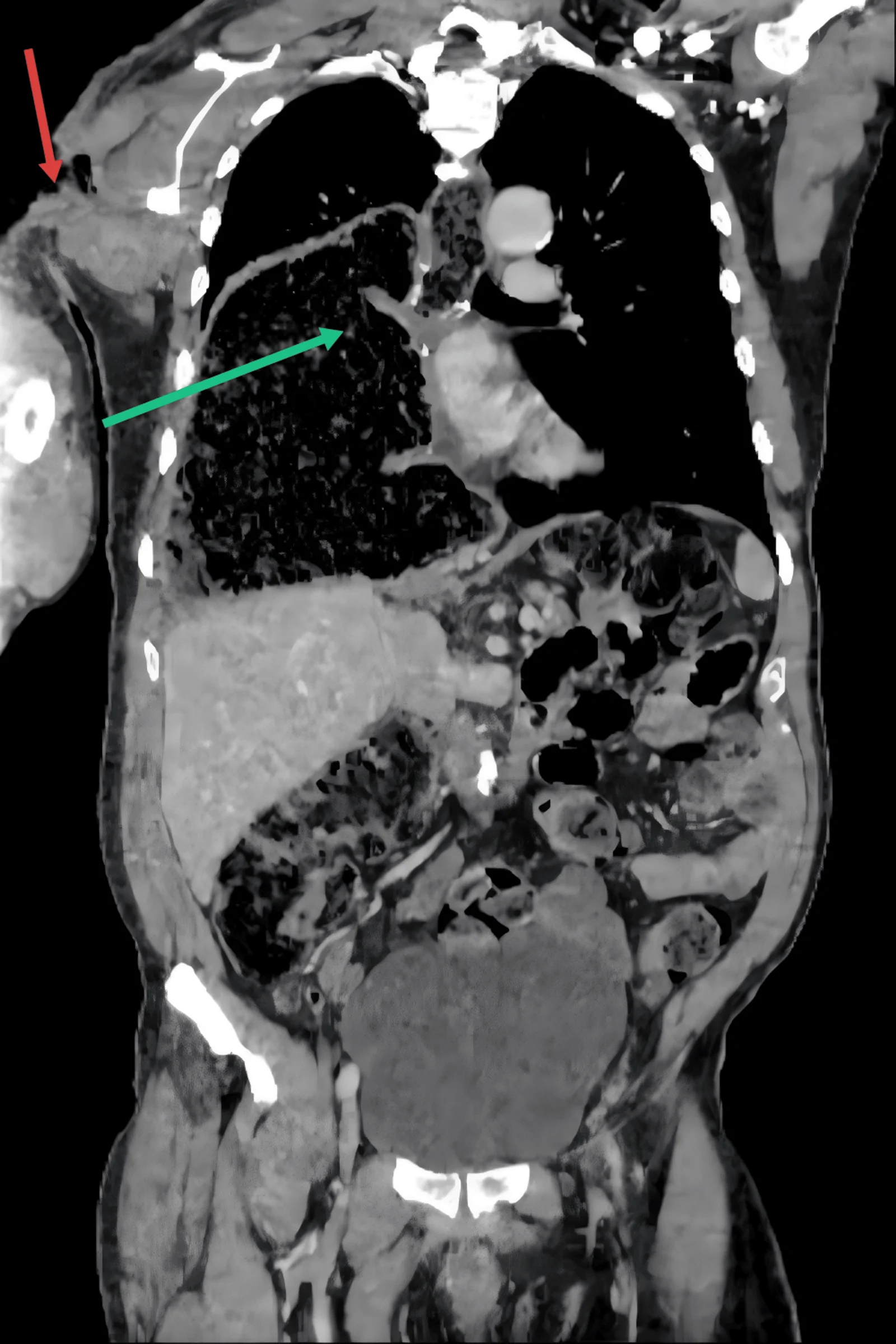
CT of the thorax, abdomen, and pelvis demonstrating postoperative changes and gas (red arrow) within the right posterior shoulder soft tissues. No pulmonary parenchymal abnormality or evidence of disseminated infection is identified. Note postesophagectomy anatomy with gastric pull-up (green arrow) CT: computed tomography

CT TAP performed on postoperative day 11 after the initial procedure demonstrated expected postoperative changes in the right shoulder, including postoperative gas within the soft tissues. No significant pulmonary abnormality or evidence of disseminated infection was identified. Postesophagectomy anatomy was confirmed, including a dilated gastric pull-up with narrowing at the diaphragmatic hiatus, consistent with known surgical history and without evidence of active pathology (Figure 3).

**Figure 4:**
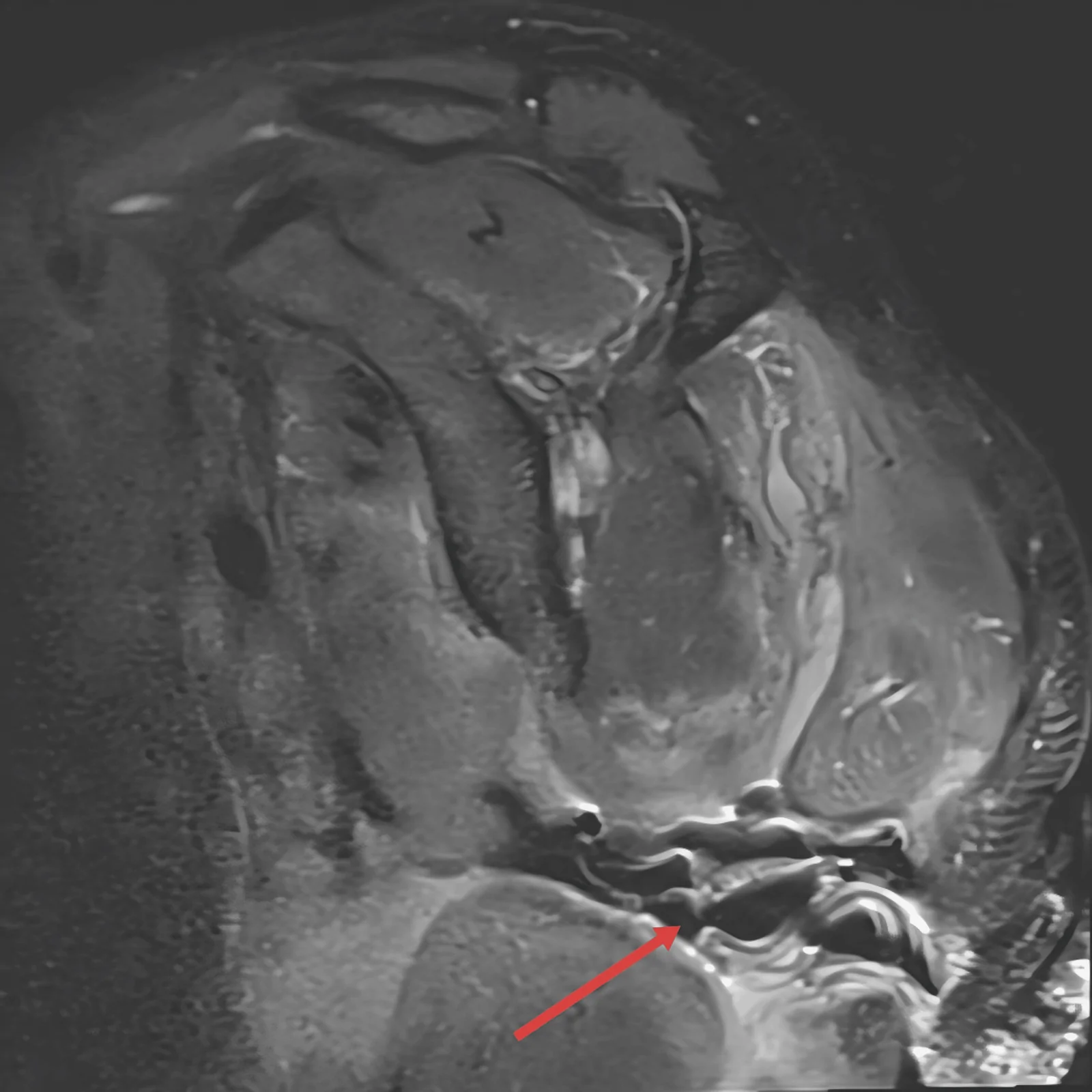
Repeat MRI of the right shoulder on postoperative day 12 from initial surgery, demonstrating a large surgical cavity (arrow) within the quadrilateral space between the teres minor and teres major muscles with adjacent reactive myositis in the T2 axial view MRI: magnetic resonance imaging

Repeat MRI of the right shoulder performed on postoperative day 12 from the initial surgery demonstrated a large surgical cavity within the quadrilateral space, positioned between the teres minor and teres major muscles and bulging anteriorly in proximity to the thoracodorsal artery. Reactive myositis was present in adjacent muscle bellies. There was no evidence of osteomyelitis, glenohumeral joint involvement, or rotator cuff disruption. The proximity of the cavity to the quadrilateral space raised awareness of potential quadrilateral space syndrome as a complication of the recovery course (Figure 4).

At review on postoperative day 13, the patient was comfortable at rest with satisfactory pain control and improving right shoulder range of movement. Neurovascular status remained intact. Transition from intravenous to oral antimicrobial therapy was initiated with co-trimoxazole 960 mg twice daily for a planned six-month course as per infectious diseases recommendations, with regular renal function monitoring. Wound care by scheduled dressing changes and pack renewal every 48 hours was arranged, with outpatient follow-up planned within one week.

## Discussion

*N. farcinica* is distinguished from other members of the *N. asteroides* complex by its greater inherent antimicrobial resistance and well-documented capacity for hematogenous dissemination, including to the central nervous system and soft tissues [1,3]. Although classically regarded as an opportunistic pathogen in immunocompromised individuals, primary cutaneous and soft-tissue nocardial disease may occur in immunocompetent hosts following direct environmental inoculation [1,2].

Musculoskeletal nocardiosis is rare and most frequently arises through hematogenous seeding from a primary pulmonary focus, with reported manifestations including pyomyositis, septic arthritis, osteomyelitis, bursitis, and deep soft-tissue abscess [4,5]. The present case is notable in that infection was localized to the deep posterior shoulder musculature, principally the teres major, without evidence of glenohumeral joint involvement, osteomyelitis, or an identifiable pulmonary source. Published cases of primary cutaneous or soft-tissue nocardiosis in immunocompetent hosts typically describe infections of the extremities following penetrating trauma, thorn injuries, insect bites, fractures, or environmental contamination [2,6-8]. A deep-space posterior shoulder infection arising after sports massage, in the absence of a documented skin breach or conventional traumatic inoculation, represents a potentially novel clinical scenario. While remaining speculative in the absence of environmental or practitioner sampling, these proposed mechanisms, namely, that vigorous deep soft-tissue manipulation either facilitated direct inoculation through an occult microscopic cutaneous injury or produced local tissue disruption/contusion that predisposed the region to secondary colonization, represent theoretical hypotheses derived from the observed temporal association rather than demonstrated causation.

Diagnosis of nocardial soft-tissue infection presents a significant clinical challenge because the presenting features of pain, swelling, erythema, systemic inflammatory response, and elevated blood markers are nonspecific and closely resemble those of more common pyogenic infections caused by *Staphylococcus aureus* or Streptococcal species [2,3]. Empirical treatment with intravenous flucloxacillin was entirely appropriate in this case given the initial presentation. The critical signal prompting microbiological reassessment was the persistence of markedly elevated inflammatory markers and ongoing systemic signs despite adequate initial surgical drainage and appropriate empirical antibiotics. This underscores a fundamental clinical principle: when a deep soft-tissue infection follows an atypical course or fails to respond as anticipated, operative cultures must be reviewed critically and extended incubation considered to identify slow-growing or atypical organisms.

A further diagnostic consideration is the growth characteristics of Nocardia species, which are slow-growing relative to routine bacterial pathogens and may require prolonged microbiological incubation and specific laboratory awareness to avoid premature culture disposal or misidentification [1,3]. Modern identification platforms, including matrix-assisted laser desorption/ionization time-of-flight mass spectrometry and molecular sequencing techniques, have significantly improved species-level recognition and the detection of uncommon pathogens such as *N. farcinica* [1,3]. Absence of clinical suspicion may therefore contribute to underdiagnosis, particularly in immunocompetent patients without classic risk factors.

Management of musculoskeletal nocardiosis requires a combination of adequate surgical source control and prolonged targeted antimicrobial therapy. Surgical drainage is an essential component because antimicrobial penetration into loculated collections is limited [4,5]. In this case, two sequential drainage procedures were required to achieve adequate source control, reinforcing the need for repeat surgical assessment when persistent cavity formation is suspected. Antimicrobial selection must be guided by species identification and susceptibility testing. Co-trimoxazole (trimethoprim-sulfamethoxazole) remains the conventional first-line agent for most nocardial infections; however, *N. farcinica* exhibits variable susceptibility profiles and may be resistant to multiple antimicrobial classes [1,3]. Linezolid shows favorable activity against many nocardial isolates, with excellent tissue penetration, and represents an important therapeutic option, particularly for resistant isolates [3]. The sequential approach in this case, transitioning from empirical intravenous flucloxacillin to targeted intravenous linezolid and subsequently to prolonged oral co-trimoxazole, is consistent with established management principles. Given the propensity of *N. farcinica* for hematogenous dissemination, cross-sectional imaging to exclude pulmonary, intra-abdominal, and central nervous system involvement is recommended [1,3]. The absence of disseminated disease on CT TAP supported management as a localized infection.

The absence of active immunosuppression in this patient warrants comment. Although the patient had a prior diagnosis of esophageal carcinoma treated by curative resection three years before presentation, formal immunosuppression screening returned negative results, and no evidence of active malignancy, chronic corticosteroid use, or other recognized immunocompromising condition was identified. This observation reinforces the principle that nocardial infection, including with *N. farcinica*, should not be excluded solely on the basis of apparent immunocompetence.

Several limitations of this report should be acknowledged. Definitive proof that sports massage directly caused nocardial inoculation cannot be established, and the temporal association between massage and symptom onset is circumstantial rather than causal. Environmental sampling of the massage setting was not performed, and an occult skin breach may have occurred without being recognized at the time of the procedure. The possibility of hematogenous seeding from an undetected pulmonary or cutaneous focus, while considered unlikely given the clinical and radiological findings, cannot be entirely excluded.

## Conclusions

This case describes an unusual and potentially novel presentation of *N. farcinica* musculoskeletal infection manifesting as a large deep posterior shoulder abscess in an immunocompetent host without an established portal of entry beyond temporal association with vigorous sports massage. The case highlights the importance of maintaining a broad differential diagnosis for deep soft-tissue infections and, specifically, of considering atypical pathogens such as Nocardia species when an infection follows an unexpected clinical course or fails to respond to conventional empirical antimicrobial therapy.

Successful management required timely surgical source control over two operative interventions, accurate species-level microbiological identification, and prolonged susceptibility-guided antimicrobial therapy within a multidisciplinary framework involving orthopedic surgery, infectious diseases, and microbiology. This report adds to the limited published literature on musculoskeletal nocardiosis in immunocompetent hosts and proposes the hypothesis that vigorous deep soft-tissue manipulation could represent a plausible, temporally associated antecedent context to consider when evaluating unexplained, localized soft-tissue infections.
